# The Need for IRB Leadership to Address the New Ethical Challenges of Research with Highly Portable Neuroimaging Technologies

**DOI:** 10.1017/jme.2024.156

**Published:** 2024

**Authors:** Donnella S. Comeau, Benjamin C. Silverman, Mahsa Alborzi Avanaki, Susan M. Wolf

**Affiliations:** 1: HARVARD MEDICAL SCHOOL, BOSTON, MA, USA; 2: BETH ISRAEL DEACONESS MEDICAL CENTER, BOSTON, MA, USA; 3: UNIVERSITY OF MINNESOTA, MINNEAPOLIS, MN, USA

**Keywords:** IRB, Research Ethics, Research Oversight, Portable MRI Research, Citizen Science

## Abstract

The emergence of innovative neuroimaging technologies, particularly highly portable magnetic resonance imaging (pMRI), has the potential to spawn a transformative era in neuroscience research. Resourced academic institutional review boards (IRBs) with experience overseeing traditional MRI have a special role to play in ethical governance of pMRI research and should facilitate the collaborative development of nuanced and culturally sensitive guidelines and educational resources for pMRI protocols. This paper explores the ethical challenges of pMRI in neuroscience research and the dynamic leadership role that IRBs should play to promote ethical oversight of emerging pMRI research.

## Introduction

We are currently on the verge of a transformative era in neuroscience galvanized by a century of advances in computing, artificial intelligence (AI),^1^ and diagnostic and therapeutic imaging.^2^ The emergence of highly portable magnetic resonance imaging (pMRI) has the potential to transform neuroimaging research^3^ and impact the medical system as a macro-innovation. A macro-innovation is defined by Flessa and Huebner as a technology that challenges “…all stakeholders, structures, processes and paradigms of the health care sector.”^4^ Emerging highly portable neuroimaging technologies, particularly ultra-low-field magnetic resonance imaging (MRI) scanners, present opportunities to transform the landscape of neuroscience by expanding research into new field settings and reaching populations currently underrepresented in neuroimaging research. They also offer opportunities for new researchers — including those outside of established research institutions and citizen scientists — to engage in neuroimaging research, due to the safety profile and reduced cost of low field pMRI scanners. Indeed, some pMRI devices may be produced non-commercially using open-source technology and 3D printers for as little as $21,000.^5^

This shift presents challenges for ethical oversight, particularly when imaging moves outside of the typical academic MRI suite and beyond institutions with established institutional review boards (IRBs) and safety committees. The absence of standard pMRI safety procedures, as well as pMRI’s expected dependence on data cloud transfer and AI processing, exacerbates old risks related to magnet safety and introduces new risks related to data security, privacy, AI bias, and the detection and management of incidental findings. The significant ethical challenges posed by this technology necessitate a transformation in research ethics governance and technology literacy.^6^

IRBs need to strengthen their expertise to handle a new generation of protocols involving community-engaged field research with a range of pMRI scanners, integrated AI, and cloud storage, often in remote and underserved populations. However, IRBs need to go further. Well-resourced IRBs in academic settings with established expertise in overseeing MRI research need to serve as a resource for external researchers and their participants.

To support this transformation, we are calling for a paradigm shift in the operations and focus of IRBs concerning internal and external research oversight and collaboration. This shift involves changes in research ethics governance, addressing existing gaps by making necessary content adjustments and engaging in bi-directional learning with communities. A significant aspect of this shift is to extend IRB oversight to external research and actively implement community engagement practices. This shift could begin in academic IRBs due to their community commitment, with private IRBs potentially following suit on community engagement. However, funding constraints could represent a major obstacle for external researchers even if resources for access to academic and private IRB review are expanded. Additionally, since IRBs vary in their expertise on MRI research and issues such as radiation safety, better-resourced institutions should recognize their ethical obligations to serve the wider human subjects research community by building the capacity to collaborate with all stakeholders to develop educational resources and ethical frameworks that facilitate safe and ethically sound pMRI research for less experienced and less resourced investigators.

This paper describes the critical role that IRBs could play in this paradigm shift. While there may be other oversight institutions and stakeholders, we believe that IRBs are particularly vital.^7^ IRBs need to strengthen their expertise to handle a new generation of protocols involving community-engaged field research with a range of pMRI scanners, integrated AI, and cloud storage, often in remote and underserved populations. However, IRBs need to go further. Well-resourced IRBs in academic settings with established expertise in overseeing MRI research need to serve as a resource for external researchers and their participants. This will challenge IRBs to reach beyond the walls of their institutions to help ensure the ethical integration of pMRI into neuroscience research. This is an opportunity for bi-directional learning — IRBs will not only share their established expertise but will also learn from new researchers and communities.

## I. Emerging pMRI Technologies

Multiple characteristics of pMRI technologies will pose challenges for IRBs, even those IRBs accustomed to overseeing protocols involving traditional MRI. The field variability of pMRI systems affects mobility, cost, device footprint, safety, power requirements, imaging protocols, image quality, and their reliance on AI for image acquisition, enhancement, and interpretation. These differences influence site deployment, training requirements for staff, and study risks. Considering the range of emerging pMRI scanners elucidates the need for ethics expertise to address issues of data privacy, informed consent, and safety monitoring, including management of incidental findings.

Irrespective of mobility, low-field and high-field MRI scanners differ significantly in how they acquire images. This difference stems primarily from the strength of the static magnetic field, which affects various aspects of the imaging process, including the quality of the images acquired and the types of applications for which the MRI can be used. Arnold et al. define ultra-low-field (ULF) as ≤ 0.01T, very-low-field (VLF) as < 0.1T, low-field (LF) as ≤ 0.3T, mid-field (MF) as ≤ 1.0T, high-field (HF) as ≤ 3T, very-high-field (VHF) as < 7T, and ultra-high-field (UHF) as ≥ 7T.^8^ Since 2018, the U.S. Food and Drug Administration (FDA) has cleared the Hyperfine Swoop head scanner (0.064T), Promaxo prostate scanner (0.066T), Synaptive Evry intraoperative scanner (0.5T), Siemens Magnetom Free Max general purpose scanner (0.55T), and Aspect Embrace neonatal scanner (1T).^9^ Additionally, the first open-source MRI scanner, the OSI^2^ ONE (~0.05T), capable of imaging the head and extremities, was built in 2022 from mostly open-source hardware and open-source software parts.^10^

The ultra-low-field Hyperfine Swoop Portable MR Imaging System^11^ was FDA-cleared in 2020 for brain imaging for patients of all ages. It is currently being introduced into academic clinical and research settings by health care professionals previously trained on high-field MRI. This portable system allows for point-of-care MRI acquisition in settings where high-field MR imaging was not previously possible, such as neonatal and neurological intensive care units, emergency departments, and operating rooms. The safety profile for ultra-low-field MRI holds the potential to move MRI out of hospitals into the community and remote field settings. Indeed, research groups have already demonstrated the feasibility of outfitting a van with pMRI for mobile and field-based research.^12^

## II. Ethical Challenges Posed by pMRI Research

Since ultra-low-field MRI scanners’ safety profile, lower cost, and smaller footprint make portability easier, these pMRI systems are likely to be widely utilized for research beyond traditional hospital and academic settings. This expansion will facilitate neuroscience research in diverse populations and environments. From a governance standpoint, understanding the issues related to field strength, mobility, and safety is an important component of oversight and necessary information for researchers, IRBs, community partners, and research participants.

The recent consensus paper by Shen and colleagues on the ethical issues raised by pMRI research detailed core challenges for IRBs, including the need to consider oversight and education of pMRI investigators and participants outside of conventional research institutions (see **Table 1**).^13^ Research institutions with the financial means to afford MRI scanners for human subjects research typically have allocated resources to ensure that neuroimaging research is conducted safely, with appropriate institutional oversight and compliance with rules and guidance promulgated by the FDA, funding agencies, and others.^14^ Practically, the prohibitive cost of conventional MRI scanners, institutional oversight requirements, and certificates of need related to hospitals obtaining MRI scanners have served to significantly limit access to MRI scanners for research.^15^ However, pMRI developers have lowered the cost and increased the accessibility of MRI by facilitating improved portability of MRI magnets of variable field strengths.^16^ Widened accessibility will require expanded training for the safe and ethical use of pMRI in neuroscience research with consideration of how to ensure appropriate oversight when research is undertaken by those outside of conventional research institutions. Examples of this may include a community group or otherwise unaffiliated researchers without formal research oversight infrastructure or a psychology professor in a small college that receives no federal grant funding.

**Table 1:** Summary of Consensus Recommendations Related to IRBs in Portable MRI Research. Reprinted and summarized from Shen et al. (2024)* with permission.

#	Operational, ethical & legal challenges for IRBs	Recommended solutions
1	**“Challenge:** Researchers and scanner operators without prior training in neuroimaging may utilize portable MRI without sufficient operational and safety training.”	Require all members of the research team to demonstrate their competency to carry out their research roles, e.g., provide evidence of previous successful MRI research, completion of relevant education, and training.
3	**“Challenge:** The rapid development of portable MRI may result in research that is overseen by an IRB not yet familiar with the issues raised by portable MRI research.”	“[E]xpert stakeholders such as MRI innovators and professional associations should develop new training resources for IRB personnel such as virtual courses on portable MRI and guidance for multidisciplinary protocol review.”
4	**“Challenge:** The accessibility of portable MRI research will invite more research that is outside IRB purview, including citizen science and industry research.”	“Where IRB review is not already required, researchers should establish a gatekeeping mechanism such as seeking private [commercial] IRB review and/or equivalent community-based review so that the research is conducted with oversight guided by the Common Rule and FDA regulations. Use of portable MRI devices in research should be restricted to those entities and individuals who can adhere to relevant FDA and professional society (e.g., ACR) guidance on MRI safety and operation standards.”
5	**“Challenge:** Researchers using portable MRI research in remote and under-resourced settings may engage in extractive, helicopter research practices.”	“Require researchers to address community engagement in their protocol.” “For studies that are monitored by an IRB, the IRB’s initial and continuing reviews [should] provide mechanisms for ensuring that researchers implement these recommended solutions.” “Research teams should be composed of individuals from diverse backgrounds, and should meaningfully engage community members prior to, during, and after the research project.”
7 & 8	**“Challenge:** The portable MRI scanning site may not provide sufficient privacy to the person being scanned” and could “be conducted in an unsafe manner due to improper setup and operation in a community setting outside the hospital.”	“Require researchers to set up a scanning environment that ensures privacy and security and develop a consent process to protect the physical and informational privacy of participants.” “Safety guidelines and education should be created by ACR, ISMRM [International Society for Magnetic Resonance in Medicine], and other professional bodies, for use of highly portable MRI in field settings.”
10	**“Challenge:** Researchers using portable MRI may not know how to interpret the data and scans.”	“Require research teams to have the requisite expertise to interpret the scans for research purposes.”
13 & 14	**“Challenge:** Researchers…may not have adequate resources to meet the demands of secure data management” and “Incidental findings (IFs) may be identified in participants who are geographically remote from health care facilities and may face barriers to accessing clinical work-up and care.”	Require that “a plan for responsible management of acquired MRI data be developed by the research team before data collection begins. Adequate resources should be in place to ensure safe and secure data acquisition, de-identification, storage, sharing, and compliance with applicable policies such as NIH data sharing policy.” “Require researchers to address management of IFs and research results of potential clinical concern” and create a pathway to timely clinical evaluation and care.

* F.X. Shen et al., “Ethical, Legal, and Policy Challenges in Field-Based Neuroimaging Research Using Emerging Portable MRI Technologies: Guidance for Investigators and for Oversight,” *Journal of Law and the Biosciences* 11, no. 1 (2024): lsae008, https://doi.org/10.1093/jlb/lsae008.

Small colleges may have an IRB but may lack sufficient expertise in areas such as radiation safety or artificial intelligence. These institutions may need guidance in the biomedical sciences and may benefit from consulting with or using more experienced IRBs. Although the community of citizen scientists in the United States is currently small, there is the potential for this technology to expand it significantly. As pMRI becomes more prevalent in the community and is increasingly used there, we expect the number of people fitting into this category to grow substantially. If research using pMRI is permitted in contexts beyond the Common Rule and FDA human subjects regulations and by researchers without MRI research experience, the potential risks to research participants would be considerable.^17^ Although it is likely FDA will require oversight for pMRI scanners, human participant pMRI research currently falls outside the Common Rule when it is not federally conducted or supported by federal funding and is not conducted by personnel at an institution that has “checked the box” on its Federalwide Assurance (FWA), thereby indicating it is extending the Common Rule and the Office for Human Research Protections’ (OHRP) compliance oversight to all of its research protocols regardless of the funding source.^18^ In these situations, IRB review may still be required if the human participant pMRI research is subject to FDA regulations. When research on pMRI is intended to evaluate the safety or effectiveness of pMRI in the “diagnosis…, cure, mitigation, treatment, or prevention of disease in [humans] or other animals, or … affect[s] the structure or any function of the body of [humans] or other animals,” the pMRI device would meet the FDA definition of a medical device and would be subject to the FDA medical device regulations.^19^ In these situations, IRB review would be required under the FDA rules on Investigational Device Exemption (IDE), both in cases of “significant risk” and when the IRB is needed to agree or disagree with a finding of “nonsignificant risk.”^20^ Based on FDA guidance on MRI research studies,^21^ most pMRI studies are likely to be in the category of nonsignificant risk medical device studies, necessitating adherence to FDA’s “abbreviated” requirements at 21 C.F.R. § 812.2(b) that include the need for IRB review.^22^

On the other hand, if the pMRI medical device is being used in a research study on-label, an IDE application would not be required. While such a study would still be considered FDA-regulated and may require physician ordering, it would not necessarily require IRB review if conducted outside of the Common Rule. Further, there will be some situations where the use of pMRI in research will not meet the FDA definition of a medical device. For example, if the pMRI is used as an instrument to study human physiology (i.e., basic physiological research), but is not being investigated for its safety or effectiveness in intervening in a disease and does not impact the structure or function of the body, the pMRI would not meet the FDA definition of a medical device, and the IDE regulations would not apply.^23^ In such instances, the pMRI study would not be considered FDA-regulated, and no IRB review would be required when the research is conducted outside of the Common Rule.

Situations that do not require IRB oversight pose increased potential risks to participants due to the lack of required independent oversight of the investigators or their research. There is a heightened risk of inadequate investigator training, qualifications, study design, and participant monitoring. Unaffiliated researchers without standard research ethics training or regulatory oversight will need to be educated on the essential requirements for the proper collection, analysis, and management of neuroimaging data. Other core issues of concern include site safety, privacy issues, informed consent, bias identification and mitigation, and incidental findings recognition and management.

While low-field pMRI systems have improved safety profiles compared to high-field strength magnets, and the serious potential physical risks associated with projectile effects are essentially negated, these low-field systems may still pose physical risks to subjects with pacemakers or other implants. Additionally, variable strength portable systems will still require physical boundaries and knowledgeable operators to understand the safe operational requirements of diverse systems. Conducting pMRI work in novel sites without established physical barriers or data transfer safeguards increases the potential risks for privacy breaches and violation of confidentiality. Even in pMRI research, participants may experience psychological stress related to the scanning process such as claustrophobia, the physical discomfort in scanning, and the need to minimize motion. Perhaps most challenging in field research with pMRI far from an established medical center will be responsible management of incidental findings. When scans are conducted in remote settings with data transferred to cloud storage and interrogated using AI, timely radiological review for incidental findings and building a pathway to clinical care including for urgent findings may be difficult.^24^ This is a challenge for researchers and IRBs within a research institution, but an even greater challenge for external researchers. Conducting research without appropriate ethical oversight may thus result in unsafe procedures that harm individuals and undermine public trust.

IRBs are essential to ensuring that affiliated researchers can perform these functions. However, IRBs are also uniquely situated to support unaffiliated external researchers, their community advisory boards (if any), and the participant population involved in the research. The consensus paper from Shen et al.^25^ offers a list of concerns and recommended solutions relevant to IRB review, both for affiliated and external researchers (see **Table 1**). Their recommendations show the necessity of oversight for pMRI research, and the serious challenges posed where there is no established oversight mechanism. Their recommendations emphasize the need for IRB governance that can effectively evaluate pMRI research by affiliated researchers, as well as to provide guidance for external researchers and institutions. They describe the need to ensure that research team members have the necessary training to competently and ethically conduct pMRI research and the expertise to accurately interpret the scans and manage incidental findings. Effective management will require adequately resourced IRBs with new training opportunities for IRB personnel and researchers that will likely be developed in partnership with MRI innovators and professional associations.

## III. The Role of IRBs

IRBs have a pivotal role to play in the safe and ethical use of pMRI in research. As the key entities charged by the Common Rule and FDA regulations with overseeing research with human participants, many IRBs — especially in academic health centers — already have general experience with the ethical issues raised by traditional MRI research. Indeed, some are currently overseeing the pilot studies related to pMRI use. While pMRI research poses more challenges than traditional MRI research, IRBs have crucial foundational knowledge. This is especially true of well-resourced IRBs in academic institutions with medical centers. It is these IRBs that can provide the resources to new researchers in this field, whether those researchers are affiliated with the IRB’s institution, are unaffiliated and supported by less resourced IRBs, or are outside the reach of Common Rule oversight in nonmedical or community groups.

### A. The Challenge for IRBs in Overseeing pMRI Research by Affiliated Investigators

When pMRI research is conducted by affiliated researchers supported by federal funding or in an institution that has executed a broad FWA, the institution’s IRB(s) will oversee informed consent, site safety, data protection and confidentiality, risk minimization, incidental findings management, and regulatory compliance. Shen et al., however, point out that even IRBs familiar with traditional MRI research may not be familiar with the additional issues posed by pMRI research, which will likely require collaboration with more experienced IRBs and additional resources and training to build up proficiency. ^26^ Both IRBs and researchers will need to be educated on the nuances of variable field strength MRI and portability-related challenges.

Currently the safety and privacy issues posed by both traditional MRI and pMRI research are addressed in well-resourced institutions by licensed MRI technologists and radiologists. However, the increased accessibility of pMRI will likely require that safety procedures and credentialing be developed by state licensing agencies and professional boards to train non-imagers to perform these tasks. Additionally, IRBs will need to ensure that institutions develop the means to manage the secure transfer, storage, and analysis of imaging data to ensure privacy, proper report generation, and screening for incidental findings. This will require research institutions to scale up management of large volumes of heterogeneous imaging data. Infrastructure to securely transfer the data from outside of institutional firewalls will need to be established as well as the means to incorporate the data into the Picture Archiving and Communication System. Due to the lower image quality of low-field MRI, AI image augmentation and postprocessing will also likely be required to more accurately analyze and interpret the lower resolution imaging findings. The infrastructure for cataloging and reporting pMRI findings will also need to be established not only to ensure the responsible management of the data and findings, but also to maintain compliance with NIH Policy for Data Management and Sharing.^27^

### B. The Opportunity for IRBs to Guide pMRI Research by External Investigators

The use of pMRI for neuroscience research performed by external researchers is likely to be subject to the same state and federal regulations for safe pMRI operation that apply to affiliated researchers. As described above, FDA regulations will often apply to pMRI research and will require IRB review. This will require that external researchers either collaborate with academic institutions with IRBs or engage a commercial IRB in order to ensure compliance with the Common Rule and FDA regulations. To meet this need, established IRBs should build capacity to provide guidance and/or oversight to unaffiliated collaborators. By doing so, established IRBs can serve as facilitators of the democratization of bioethical oversight of neuroscience research using pMRI.

Meeting the needs of external pMRI researchers will pose challenges to established IRBs due to limited resources, operational obstacles, and liability concerns, among other factors. Many academic IRBs are already under-resourced and struggle to keep up with the volume of research requiring their review.^28^ Because of resource limitations, such IRBs may have already made the decision not to provide oversight for external sites or researchers, for example, by ceding review to other IRBs when single IRB review is mandated under the Common Rule. ^29^ On a practical level, external researchers may be unable to access software systems needed to submit protocols to academic IRBs. While commercial IRBs may offer a solution to these problems and would likely be the best fit for unaffiliated researchers, the researchers may not have a budget large enough to cover the associated costs.

At the same time, external researchers, including community researchers and citizen scientists, may be skeptical of IRBs due to concerns about cost, over-regulation, and slowing the pace of their work.^30^ Yet, as described above, external researchers will in many circumstances need IRB review, due to FDA regulations, the Common Rule, or state laws that may be related to operation of MRI scanners in nontraditional, “public” spaces such a gyms or community centers. Established IRBs have the opportunity to work collaboratively with external researchers to help educate them about these requirements. By fostering a clear understanding of the steps needed to protect human subjects, IRBs can effectively guide external researchers — including community researchers and citizen scientists — in conducting pMRI studies that adhere to high ethical standards, ensuring that the well-being and rights of participants are protected.^31^

To address the skepticism that some citizen scientists in particular have of IRBs, it will be essential to focus on enhancing understanding, transparency, and adaptability within the IRB framework to better accommodate the unique aspects of citizen science. IRBs need to adapt their ethical oversight to recognize the diverse formats, goals, and strengths of citizen science projects. They can address skepticism by developing guidelines that reflect the participatory nature of citizen science, acknowledging the value of individuals’ contributions to scientific research, and ensuring that these contributions are ethically managed. Moreover, addressing ethical issues in citizen science, such as potential failures of informed consent and data management concerns, is crucial. IRBs can mitigate skepticism by explicitly recognizing these issues, engaging respectfully with citizen scientists to understand the issues posed by their research, and developing clear guidance on how to navigate those issues. IRBs could benefit from fostering open communication with citizen scientists. This might include offering workshops to foster communications with the citizen science community.^32^ IRBs can help facilitate the development and exchange of resources ranging from web-based materials to development of multi-stakeholder ethics working groups, depending on local community needs. Dynamic collaboration with community groups and citizen scientists is also likely to provide tangible societal value to the research.

### C. The Need for Community Engagement & Bi-Directional Learning for Research Ethics

Shen et al. emphasize that pMRI research is particularly helpful in reaching underserved and remote populations. It is essential to recognize that meaningful research efforts in these areas necessitate active community engagement to ensure both relevance and respect in the process.^33^ While academic and commercial IRBs may be best positioned to lead oversight of the next generation of pMRI research, a narrow, stagnant view of their responsibilities will not allow them to meet the oversight challenges of community-engaged and democratized pMRI research. In addition to their traditional review roles, IRBs should work to expand their purview to include the assessment of the societal impact of pMRI, an AI-augmented macro-innovation. They should also build the capacity to provide educational resources to external researchers and community stakeholders, including those involved in community-engaged, community-led, and community-based participatory research. In some cases, they may be asked to conduct a review of research proposed by external researchers; IRBs should work with their institution in an effort to grant such requests where feasible.

IRBs have the opportunity to be leaders working with researchers and sponsors to promote transparency and to incorporate diverse voices into ethical governance for pMRI technologies. Not all researchers and communities will welcome IRB involvement, as some may mistrust IRBs based in academic institutions due to past unethical practices in research, unevenness in IRB quality, and critiques of IRBs and the oversight system for research with human participants.^34^ IRBs will need to address these issues and demonstrate their quality, utility, and commitment to ethical oversight of community-based research. They will need to develop a transparent and equitable approach in partnership with all stakeholders, while simultaneously building trust through consistent, ethical practices and inclusive, community-centered consultation. This includes incorporating community perspectives in partnerships with shared decision-making power and removing existing IRB obstacles. These steps are essential to evolving the roles of IRBs and community partners in research. ^35^

One of the core concerns related to disruptive research technologies, specifically AI research, is the potential for societal harm. Current federal regulations governing human subjects research stipulate that IRBs must evaluate non-exempt research protocols to determine if they meet criteria for approval. Approval requires minimization of risks, that the risks are reasonable relative to the potential benefits if any, that subject selection is equitable, and that appropriate informed consent is obtained, among other requirements. However, these requirements fail to fully consider societal harms. Indeed, federal regulations specify, “[t]he IRB should not consider possible long-range effects of applying knowledge gained in the research (e.g., the possible effects of the research on public policy) as among those research risks that fall within the purview of its responsibility.”^36^ Unlike many international research ethics bodies, U.S. IRBs are not permitted by their governing regulations to consider societal harms in reviewing research proposals. This exclusion prevents potential long-term risks from impeding beneficial research, while also ensuring that long-term benefits do not undermine protections for research participants.^37^

Given this oversight gap, Stanford University has created an ancillary review process — an Ethics and Society Review (ESR) board — to assess risks to human society for all internally funded AI projects, rather than solely focusing on risks to human subjects.^38^ This added level of scrutiny should also be considered for other macro-innovations like pMRI. While official incorporation of ESR into the Common Rule would require a regulatory change, integrating ESR into oversight of pMRI research beyond the Common Rule would not. This could be done by creating dedicated committees like an ESR board and could be supported by expert advice from the National Academies of Sciences, Engineering, and Medicine or the Secretary’s Advisory Committee on Human Research Protections. ^39^ Incorporating ESR review — whether by regulatory change to expand the scope of IRB analysis or by focusing (at least initially) on research beyond the Common Rule — can reimagine research ethics in response to these emerging challenges. This transformation of research oversight from a narrow concern with protecting human participants in research by affiliated researchers into a more dynamic enterprise that considers broader impacts and a wider range of researchers necessitates a bi-directional learning process, with non-traditional and community researchers learning from IRBs and vice versa. This approach holds the potential to improve IRB processes for review of community-engaged research and to respond to the evolving character of neuroimaging research as field-based pMRI gains traction. Ultimately, the democratization of both brain research and research oversight, as spurred by the advent of highly portable MRI, represents a potential step forward in ethical research practices.

IRBs should also be enabled to work with researchers, sponsors, and professional societies to ensure that pathways to clinical care for participants with findings of concern are established prior to the start of a pMRI imaging study, regardless of the imaging participant’s geographic location, insurance status, and ability to pay for care. IRBs could assist in developing collaborative partnerships between professional societies, sponsors, academic medical institutions, and community researchers to ensure ethical management, including appropriate clinical follow-up of incidental findings and research results for research participants.

Effective oversight of pMRI research will require the development of operational standards and procedures through professional societies, such as the International Society for Magnetic Resonance in Medicine (ISMRM) and American College of Radiology (ACR), as well as MRI developers and industry.^40^ Safety guidelines and related educational resources should be created by radiology professional bodies for use of pMRI in field settings, just as they have created guidance for institutionally sited MRI scanning. IRBs, particularly those with experience providing MRI research oversight, can offer guidance and education related to established policies for safe setup, use, storage, and transport of the equipment and standards for participant privacy and data security. IRBs can also help develop resources to ensure that pMRI research is restricted to those entities and individuals credentialed by professional organizations to ensure adherence to relevant FDA and professional society (e.g., ACR) guidance on MRI safety and operation standards.

Some academic IRBs are already familiar with new low-field pMRI, having overseen early pilot studies. These organizations are particularly well positioned to create and share guidelines based on best practices and experiences. They should be encouraged and financially supported to collaborate with professional MRI organizations, industry, and other academic and commercial IRBs to develop educational materials that can be shared among IRBs, affiliated and external researchers, and community groups. IRBs should also be enabled to work with researchers, sponsors, and professional societies to ensure that pathways to clinical care for participants with findings of concern are established prior to the start of a pMRI imaging study, regardless of the imaging participant’s geographic location, insurance status, and ability to pay for care.^41^ IRBs could assist in developing collaborative partnerships between professional societies, sponsors, academic medical institutions, and community researchers to ensure ethical management, including appropriate clinical follow-up of incidental findings and research results for research participants.

IRBs actually have a long history of community involvement. Federal regulations require “sensitivity to such issues as community attitudes” and the inclusion of at least one member from outside the institution on the IRB roster, ^42^ and IRBs should work to include and prioritize their input. IRB knowledge gained from working with community members, for example in the development of lay-language research ethics training materials for community member onboarding, can be shared with researchers and community partners doing pMRI research. At the same time, IRBs have at times struggled with review of community-engaged research.^43^ Challenges have included genuine and sustained integration of community members, adjustment of materials to lay language and appropriate literacy levels and creating accessible and culturally competent research ethics training. IRBs should work with and learn from community partners to address these challenges. For example, IRBs can collaborate with community partners in the process of developing written research study materials such as consent forms, to ensure their appropriateness for community participants. Similarly, guidance for the ethical practice of AI and pMRI research should be developed as a collaboration between community partners, researchers, and the IRB, so that the materials can reflect the cultural diversity, local context, and priorities of the community.

## IV. Envisioning the Future of Research Oversight: Democratizing Research Ethics

The accessibility of smaller, less costly imaging devices will enable research by a broader range of investigators, including those who are less experienced, unaffiliated, or citizen scientists. These groups may conduct research beyond the reach of the Common Rule and FDA regulations, potentially with limited or no organizational oversight. In this rapidly evolving landscape, IRBs must play a central role in developing guidance to address the challenges arising from the growing use of pMRI in neuroscience research. IRBs are key to formulating oversight strategies beyond the traditional regulatory approach for research that has the potential to harm individuals and the larger public.

IRBs cannot address the oversight challenges in isolation from the realities of pMRI research. Portable MRI research will greatly expand the roster of stakeholders, include new participants and communities historically excluded from neuroscience research, and offer research opportunities to new categories of researchers outside of traditional research spheres. To engage these new communities and stakeholders, IRBs will need to go beyond the walls of the institution and the circle of affiliated researchers to engage participant communities and external researchers. Much as pMRI promises a new era of far more democratized neuroscience research, it also demands a new vision of more democratized research oversight. IRBs have an opportunity to deepen their dialogue with communities and new researchers. The bi-directional learning required can enrich and transform research ethics and oversight, integrating new perspectives and contributing value to participants and communities.

## Conclusion

Resourced academic IRBs are well positioned to help develop the essential components of oversight for responsible pMRI neuroscience research. However, development of the necessary infrastructure will require collaboration with multiple stakeholders including less resourced IRBs, community ethics boards, federal and state agencies, professional and licensing boards, and citizen science groups to develop the necessary components. IRBs should adapt their oversight approaches to these changing contexts, accommodating the diverse range of investigators and research settings. This revision requires a deep understanding of the unique ethical and operational challenges posed by conducting pMRI research using scanners of different field strengths in field settings. IRBs are also well positioned to pioneer innovative models of oversight for community researchers who operate beyond the Common Rule. Organizations such as OHRP and Association for the Accreditation of Human Research Protection Programs could help support this effort by suggesting the minimum requirements for ethical oversight of external and internal researchers. Ethical oversight of external pMRI research should involve IRB collaboration with community oversight boards, use of ESR boards, and active engagement with external researchers and community partners for bi-directional learning to ensure responsible and just research practices.
